# Artificial Intelligence for the Prediction and Early Diagnosis of Pancreatic Cancer: Scoping Review

**DOI:** 10.2196/44248

**Published:** 2023-03-31

**Authors:** Zainab Jan, Farah El Assadi, Alaa Abd-alrazaq, Puthen Veettil Jithesh

**Affiliations:** 1 College of Health & Life Sciences Hamad Bin Khalifa University Doha Qatar; 2 AI Center for Precision Health Weill Cornell Medicine-Qatar Doha Qatar

**Keywords:** artificial Intelligence, pancreatic cancer, diagnosis, diagnostic, prediction, machine learning, deep learning, scoping, review method, predict, cancer, oncology, pancreatic, algorithm

## Abstract

**Background:**

Pancreatic cancer is the 12th most common cancer worldwide, with an overall survival rate of 4.9%. Early diagnosis of pancreatic cancer is essential for timely treatment and survival. Artificial intelligence (AI) provides advanced models and algorithms for better diagnosis of pancreatic cancer.

**Objective:**

This study aims to explore AI models used for the prediction and early diagnosis of pancreatic cancers as reported in the literature.

**Methods:**

A scoping review was conducted and reported in line with the PRISMA-ScR (Preferred Reporting Items for Systematic Reviews and Meta-Analyses Extension for Scoping Reviews) guidelines. PubMed, Google Scholar, Science Direct, BioRXiv, and MedRxiv were explored to identify relevant articles. Study selection and data extraction were independently conducted by 2 reviewers. Data extracted from the included studies were synthesized narratively.

**Results:**

Of the 1185 publications, 30 studies were included in the scoping review. The included articles reported the use of AI for 6 different purposes. Of these included articles, AI techniques were mostly used for the diagnosis of pancreatic cancer (14/30, 47%). Radiological images (14/30, 47%) were the most frequently used data in the included articles. Most of the included articles used data sets with a size of <1000 samples (11/30, 37%). Deep learning models were the most prominent branch of AI used for pancreatic cancer diagnosis in the studies, and the convolutional neural network was the most used algorithm (18/30, 60%). Six validation approaches were used in the included studies, of which the most frequently used approaches were k-fold cross-validation (10/30, 33%) and external validation (10/30, 33%). A higher level of accuracy (99%) was found in studies that used support vector machine, decision trees, and k-means clustering algorithms.

**Conclusions:**

This review presents an overview of studies based on AI models and algorithms used to predict and diagnose pancreatic cancer patients. AI is expected to play a vital role in advancing pancreatic cancer prediction and diagnosis. Further research is required to provide data that support clinical decisions in health care.

## Introduction

### Background

Pancreatic cancer is usually associated with poor prognosis, and the 5-year survival rate is less than 6% [1]. It is the 7th leading cause of mortality among all cancer types and the 12th most common cancer across the globe [1,2]. The main cause of a poor prognosis is a late diagnosis. In recent years, studies have focused on finding biomarkers that potentially contribute to early diagnosis, lowering morbidity and mortality [3]. Early diagnosis of pancreatic cancer could improve patient outcomes with curative surgery. Hence, identifying individuals with high-risk or precursor lesions should be the focus of efforts to increase survival. Diagnosis of pancreatic cancer can be achieved by an efficient screening method involving ultrasonography, some biological markers, or the family history of patients [4]. By 2030, pancreatic cancer is expected to be the primary cause of cancer deaths with an unprecedented high mortality rate [5]. In the absence of effective ways to diagnose pancreatic cancer at an early stage, the disease could be severely advanced by the time a standard diagnosis is initiated. Therefore, developing tools that allow early and accurate diagnosis of pancreatic cancer is vital to lowering mortality and increasing survival [5].

Artificial intelligence (AI) includes a large family of techniques to generate simplified representations from complex data that can be used for decision-making or classification [6]. AI is constantly improving the health care sector. With the growing usage of electronic health records, the advances in computer power and continuous monitoring systems and the availability of large data, AI technologies have become the ideal medium for improving health care [7]. Even though it remains expensive and unfeasible to screen the general population for pancreatic cancer with the present technology, the ability to identify susceptible populations with a higher possibility of harboring such lesions may lead to earlier interception and enhanced survival rates. The use of AI and machine learning as risk stratification tools have the potential to change the diagnosis and the diagnostic landscape. Previous studies reported that AI algorithms have benefited physicians through clinical diagnostic prediction and imaging-based testing [8,9].

### Research Problem and Objective

A huge number of articles has been published on AI technologies for pancreatic cancer. Several reviews were conducted to summarize previous studies; however, they had the following limitations. First, they focused on the diagnosis of pancreatic cancer and AI, but they did not describe in detail the features of the AI algorithms used [9]. Second, they did not search relevant databases such as Google Scholar and Science Direct [10,11]. Third, they were restricted to diagnosing pancreatic cancer using only ultrasound [10] and imaging [12]. Fourth, they focused on the application of machine learning in clinical decision support systems for the management of pancreatic cancer [11]. Last, their scope was limited to the applications of deep learning methods in the field of pancreatic cancer imaging [13]. However, the available literature lacks a review that provides an overview of AI’s application in the diagnosis of pancreatic cancer. Thus, the current review aims to explore features of AI models used for the diagnosis and prediction of pancreatic cancer to help the scientific community advance research in this field by identifying gaps and looking into future prospects.

## Methods

### Overview

In this scoping review, PRISMA-ScR (Preferred Reporting Items for Systematic Reviews and Meta-Analyses Extension for Scoping Reviews) guidelines were followed to ensure the transparency and reliability of this study. PRISMA-ScR guidelines are highly endorsed by the Joanna Briggs Institute and Cochrane for scoping reviews [14]⁠. Protocols used in the scoping reviews are detailed in the following sections.

### Search Strategy

#### Search Sources

To achieve the purpose mentioned earlier, we performed search queries between June 30, 2022, and July 30, 2022, in 5 online databases: MedRXiv, BioRXiv, PubMed, Google Scholar, and Science Direct. From Google Scholar, we retrieved a massive number of hits and ordered them based on their relevance; only the first 100 hits were included in this review. The search focused on both medical and computer science databases. To identify additional studies, we also performed backward reference list checking and forward reference list checking.

#### Search Terms

To develop the search query, 2 experts in the field of AI and digital health were consulted, and previous relevant reviews were checked. The search terms were chosen based on the target intervention (ie, artificial intelligence, machine learning, and deep learning) and the target disease (ie, pancreatic cancer). Details about the exact search terms used for searching each database are presented in Multimedia Appendix 1.

### Study Eligibility Criteria

This review included studies that focused on AI techniques that are utilized for pancreatic cancer diagnosis and risk prediction. Specifically, we focused on AI models used for any purpose related to the diagnosis of pancreatic cancer. On the other hand, this review excluded articles that summarized an overview of AI approaches for pancreatic cancer (eg, literature reviews, thesis) and studies that were purely based on clinical trials and experimental studies. We only included journal articles and conference papers, while we excluded case reports, reviews, dissertations, proposals, conference abstracts, editorials, generic, and commentaries. We excluded studies that used non-AI–based techniques for the diagnosis of pancreatic cancer. Moreover, we also excluded studies that showed a theoretical framework of AI models for pancreatic cancer. This review considered studies published only in English between January 2015 and July 2022. We applied no restrictions on the study setting, study design, study outcome, month, and country of publication.

### Study Selection

In the study selection process, we followed 3 steps. In the first step, we used Rayyan to remove the duplicates from all the retrieved studies. In the second step, screening of the titles and abstracts was done by 2 reviewers independently. Last, the reviewers independently reviewed the full text of the articles that passed the previous step. Any disagreement between the 2 reviewers was resolved by discussion. To measure the agreement between reviewers, we calculated the Cohen kappa [15], which was 0.98 for the screening of title and abstract, while it was 0.94 for the screening of the full text, showing almost perfect agreement [15]. Multimedia Appendix 2 provides the interrater agreement matrix for each reviewer.

### Data Extraction

For accurate data extraction of the included studies from the Rayyan, a data extraction table was created using Microsoft Excel and pivoted using the 30 included studies shown in Multimedia Appendix 3. Two reviewers independently performed this process, while any disagreements between the 2 reviewers were resolved through discussions. The reviewers’ agreement was 89%.

### Data Synthesis

After extracting data from the included studies, we performed data synthesis using a narrative approach. Particularly, we summarized and described the AI techniques used in the studies including their purpose (eg, diagnosis, identification, prediction, segmentation), features, data source (eg, participant and databases), and AI models (eg, support vector machine, neural network). Furthermore, we described the programming languages used for the AI techniques: data type (eg, clinical data, laboratory data, biological data) and statistical data (eg, accuracy, specificity, sensitivity, precision). Microsoft Excel was used for the management of the synthesis data. Zotero was used for reference management.

## Results

### Search Results

**Figure 1:**
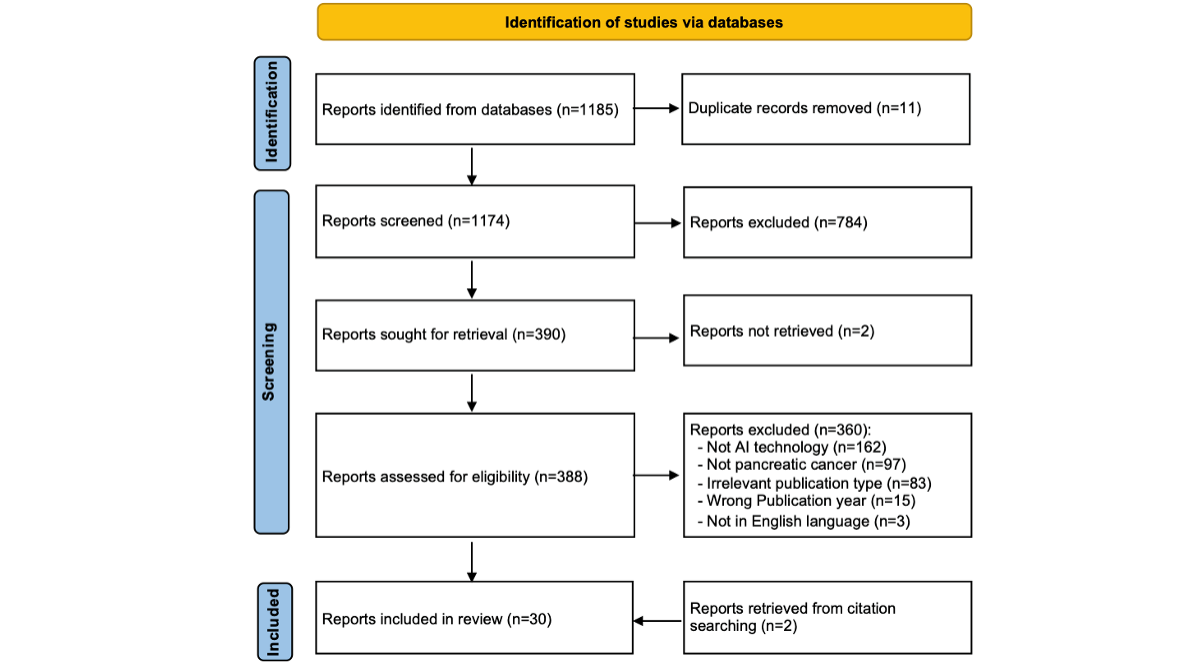
PRISMA-ScR (Preferred Reporting Items for Systematic Reviews and Meta-Analyses Extension for Scoping Reviews) flowchart of the study selection. AI: artificial intelligence.

We initially identified 18,285 articles using 5 publicly available online databases: PubMed (n=60), Science Direct (n=509), Google Scholar (n=17,200), BioRXiv (n=434), and MedRxiv (n=82). Given the huge number of articles identified from Google Scholar, we selected the top 100 articles of these articles. Hence, a total of 1185 articles were passed through the PRISMA-ScR flowchart. We excluded 11 duplicates. Of the remaining studies, 784 articles were removed after the title and abstract screening. We could not find the full text of the 2 articles, which resulted in including 388 articles for full-text screening. After reviewing the full text, we excluded 360 articles based on several reasons, as shown in Figure 1. Additionally, we identified 2 relevant articles by backward and forward-checking the reference list. In total, 30 articles met our inclusion criteria and were included in this scoping review (Figure 1).

### Main Characteristics of the Included Articles

Characteristics of the included studies are presented in Table 1. We found that all of the included studies were published in peer-reviewed journals (30/30, 100%). The included studies were published between 2015 and 2022 (Table 1), but 30% (9/30) of the studies were published in 2020. The included studies originated from 14 different countries, most of which came from the United States (8/30, 27%), followed by China (5/30, 17%). Moreover, the mean age of participants was reported in 21 studies and ranged between 18 years and 91 years, with an average of 55.6 (SD 8.3) years. Only 3 included studies involved children (≤18 years), whereas 15 included studies involved older patients (≥60 years). The number of participants mentioned in the included studies ranged from 40 to 8,110,706, with an average of 406,260.8 (SD 1,702,567) participants. The range of female participants reported in the 15 studies varied between 0% and 54.8%. More than 90% (25/30) of the included studies recruited participants with pancreatic cancer, and 3% (1/30) of the studies included both healthy participants and patients with cancer. Multimedia Appendix 4 shows the detailed characteristics of the included studies.

**Table 1:** Characteristics of the included studies (n=30).

Characteristics		Studies	References
**Publication type**			
	Journal articles	30 (100)	[16-45]
**Year of publication, n (%)**			
	2022	2 (7)	[21,30]
	2021	7 (23)	[22,23,25,32,40,42,43]
	2020	9 (30)	[16,17,20,29,34-36,41,45]
	2019	5 (17)	[19,24,28,31,37]
	2018	4 (13)	[26,27,33,39]
	2016	2 (7)	[38,44]
	2015	1 (3)	[18]
**Country of publication**			
	United States	8 (27)	[18,19,22,27,31,33,39,42]
	China	5 (17)	[24,28,34,40,41]
	India	4 (13)	[26,30,36,38]
	Iran	2 (7)	[20,21]
	Japan	2 (7)	[25,35]
	Indonesia	1 (3)	[17]
	South Korea	1 (3)	[32]
	South Africa	1 (3)	[16]
	Saudi Arabia	1 (3)	[23]
	Germany	1 (3)	[29]
	Spain	1 (3)	[37]
	United Kingdom	1 (3)	[43]
	Brazil	1 (3)	[45]
	Turkey	1 (3)	[44]
Age of participants (years), mean (SD)		55.6 (8.3)	[19,22-25,29,31-37,39-42,44]
**Age of the participants (years), n (%)**			
	≤18	3 (10)	[22,36,44]
	19-40	3 (10)	[19,23,44]
	41-60	9 (30)	[19,22,23,29,34,39,41,42,44]
	≥60	15 (50)	[19,22,24,25,29,31-37,39-41,44]
Number of participants, mean (SD)		1,702,567 (406,260.8)	[16-36,38-45]
Number of participants, range		40-8,110,706	[16-36,38-45]
**Number of participants, n (%)**			
	≤99	4 (13)	[23,28,31,36]
	100-999	18 (60)	[16,17,20,21,24,25,27,29,33-35,38,40-42,44,45]
	≥1000	6 (20)	[18,19,22,32,39,43]
	Not reported	2 (7)	[26,30]
**Gender (%), range**			
	Female	0-54.8	[22,23,25,28,29,31,32,34,36,37,39-42,44]
**Participants’ health conditions, n (%)**			
	Cancer	25 (90)	[16-22,24-38,40-42]
	Both cancer and healthy	1 (3)	[45]

### Characteristics of AI Techniques for Pancreatic Cancer

Of the included studies, 8 studies (8/30, 27%) used only machine learning algorithms, 11 studies (11/30, 37%) used only deep learning algorithms, 7 studies (7/30, 23%) applied machine learning including deep learning algorithms, and a few studies used other AI techniques such as adversarial networks in 1 study (1/30, 3%) and particle swarm intelligence in 3 studies (3/30, 10%). Moreover, different AI models, algorithms, and methods were used in the included studies for pancreatic cancer. The most commonly used models were convolutional neural networks (18/30, 60%), followed by logistic regression (6/30, 20%). In 8 studies (8/30, 27%), AI techniques were implemented based on machine learning algorithms and models. In 3 studies (3/30, 10%), AI techniques were based on particle swarm intelligence for precise and early diagnosis of pancreatic cancer. Furthermore, AI algorithms were used for 6 different purposes in the included studies, but the most common purposes were diagnosis (14/30, 47%) and risk predictions (14/30, 47%; Table 2). Only 8 studies stated the programming languages used to develop the models, and they were Python (6/30, 20%), Java (1/30, 3%), and R (1/30, 3%). Multimedia Appendix 5 shows the characteristics of the AI techniques used in each study.

As presented in Table 3, different types of data were used in the included studies: 30% (9/30) of the studies involved laboratory data (eg, patient samples, tissue samples, mRNA, blood cell count, blood samples, cancer antigens, blood cell data, and gene expression data), 40% (12/30) of the studies included clinical data (eg, patient history, disease symptoms, phenotypic data, procedures, surgeries, medications, clinical notes, BMI, and vital signs), 47% (14/30) of the studies used radiology images (magnetic resonance imaging, endosonographic, and computed tomography [CT] images), and only 3% (1/30) of the studies included demographic data (eg, age, gender, and ethnicity). In addition, 14 studies used only 1 type of data to develop their models (ie, radiological images), while the rest of the studies (16/30, 53%) used more than 1 type of data. Moreover, the number of data points stated by 28 (28/30, 93%) of the included articles ranged from 15 to 13,585,634. The included studies used data sets from either a closed source (ie, data collected directly from participants in the study or from databases in clinical settings; 26/30, 87%) or open source (ie, publicly available databases; 4/30, 13%). The numbers of features used to develop the models in the included studies ranged from 2 to 18,220, but about one-half of the included studies (16/30, 53%) did not exceed 10 features in developing their model. A detailed description of the number of features and data types used is provided in Multimedia Appendix 6.

The validation techniques used in the development of AI models in the included studies are shown in Table 4. The included studies used 5 different validation techniques, of which k-fold cross-validation (10/30, 33%) and external validation (10/30, 33%) were the most commonly used methods. Confusion matrix was only mentioned in 17% (5/30) of the included studies. The performance measures of the AI models were mentioned in 22 articles. The most commonly used performance measures were sensitivity (12/30, 40%) and specificity (12/30, 40%). As shown in Table 5, the accuracy of the AI algorithms reported in 9 studies ranged from 71.6% to 99%, with a mean of 89.4%. The sensitivity in 12 studies ranged between 60% and 99.9%, with a mean of 91.3%. The mean specificity in the 12 studies was 83.2%, with a range between 69.5% and 100%. The area under the curve (AUC) reported in 9 studies varied between 86% and 95.3%, whereas the mean was 88.05%. The minimum and maximum precision values in 3 studies were 14% and 99.5%, respectively, with a mean of 69.1% (Table 5; Multimedia Appendix 5).

**Table 2:** Types of artificial intelligence (AI) techniques used for pancreatic cancer (n=30 studies).

Types		Studies, n (%)	References
**AI type**			
	Deep learning (DL)	11 (37)	[21-23,27,33,34,36,40,42,44,45]
	Machine learning (ML)	8 (27)	[17-19,29,31,32,37,43,45]
	DL and ML	7 (23)	[16,20,25,35,38,39,41]
	Others	4 (13)	[20,26,28,30]
**AI algorithms/models/methods^a^**			
	Convolutional neural network	18 (60)	[19,21-25,27,28,33-36,38,39,41,42,44,45]
	Logistic regression	6 (20)	[16,22,25,37,39,41]
	Particle swarm optimization	3 (10)	[20,26,30]
	Random forest	2 (7)	[29,32]
	Support vector machine	2 (7)	[16,17]
	Cubic k-nearest neighbors	1 (3)	[16]
	Natural language processing	1 (3)	[18]
	Bayesian model	1 (3)	[31]
	Levenberg-Marquardt algorithm	1 (3)	[38]
	Decision tree	1 (3)	[16]
	Linear discriminant	1 (3)	[16]
	K-means clustering	1 (3)	[16]
	Adversarial network	1 (3)	[28]
	eXtreme gradient boosting	1 (3)	[43]
	Fully end-to-end DL model	1 (3)	[40]
**Purpose of AI algorithms**			
	Diagnosis	14 (47)	[16-18,20,23-26,29,32,35,40,43,45]
	Risk prediction	14 (47)	[18,20,21,26,29-31,33,35,36,38,40-42]
	Grade prediction	1 (3)	[28]
	Molecular subtype prediction	1 (3)	[29]
	Survival rate prediction	1 (3)	[41]
	Treatment response prediction	1 (3)	[31]
**Programming languages^b^**			
	Python	6 (20)	[17,23,29,39,41,45]
	Java	1 (3.3)	[18]
	R	1 (3.3)	[23]

^a^Some studies used more than one model.

^b^Only 8 studies reported the programming languages used to develop the model.

**Table 3:** Features and types of data used in the included articles (n=30 studies).

Features		Studies, n (%)	References
**Data type^a^**			
	Radiology images	14 (47)	[21,24-31,34,36,40,42,44]
	Clinical data	12 (40)	[18,19,22,23,32,33,35,37-39,41,43]
	Laboratory data	9 (30)	[16,17,20,23,32,35,39,41,45]
	Demographic data	1 (3)	[16]
Number of data points, range		15-13,585,634	[16-32,34-37,39-45]
**Number of data points**			
	<100	7 (23)	[16,26,28,30,31,34,37]
	100-999	11 (37)	[17,20,21,25,27,29,35,40,41,44,45]
	>1000	10 (33)	[18,19,22-24,32,36,39,42,43]
	Not reported	2 (7)	[33,38]
**Type of data set source**			
	Closed	26 (87)	[17-19,22-31,33-44]
	Open	4 (13)	[16,20,21,45]
**Number of features**			
	1-10	16 (53)	[17,18,20-23,25-28,30,32,35,36,39,40]
	11-20	4 (13)	[19,24,33,44]
	21-30	1 (3)	[37]
	>30	6 (20)	[15,28,30,33,40,42]

^a^Many studies used more than data type.

**Table 4:** Validation approaches and performance measures (n=30 studies).

Validation and statistics		Studies, n (%)	References
**Validation approach^a^**			
	External validation	10 (33)	[18,20-22,28,31,33,37,38,40]
	K-fold cross-validation	10 (33)	[16,17,19,23-25,28,34,35,37,39]
	Hold-out cross-validation	5 (17)	[19,22,39,41,43]
	Leave-one-out cross-validation	2 (7)	[31,35]
	Shuffle-split cross-validation	1 (3)	[29]
	Not reported	4 (13)	[26,32,36,42]
**Confusion matrix**			
	Reported	5 (17)	[18,20,35,40,45]
	Not reported	25 (83)	[16,17,19,21,23-34,36-38,41,42,44]
**Performance measures^b^**			
	Sensitivity	12 (40)	[16-20,27,29,33,40,43-45]
	Specificity	12 (40)	[16-20,22,27,29,40,43-45]
	Accuracy	9 (30)	[16,17,20,28,33,34,37,40,44]
	Area under the curve	11 (37)	[16,19,25,28,29,31,32,34,40,41,43]
	Precision	3 (10)	[21,22,24]
	*F*_1_-score	1 (7)	[45]
	Mean absolute error	1 (3)	[33]
	Cohen kappa	1 (3)	[16]
	Root means square error	1 (3)	[33]

^a^Total number does not add up, as many studies used more than 1 validation method.

^b^Total number does not add up, as many studies used more than 1 performance measure.

**Table 5:** Overview of performance of artificial intelligence (AI) models.

Performance measures	Results (%), mean (range)
Accuracy	89.4 (71.6-99)
Area under the curve	88.05 (86-95.3)
Precision	69.1 (14-99.5)
Sensitivity	91.3 (60-99.9)
Specificity	83.2 (69.5-100)

## Discussion

### Principal Findings

In this study, we explored the AI techniques used for the prediction and early diagnosis of pancreatic cancer. We found a scarcity of articles in 2017; however, more studies have been published in the past 4 years, unsurprisingly due to the increasing dependence of the health care system on AI technology. Of the 1185 articles, 30 articles were included in this scoping review (5 in 2019, 9 in 2020, 7 in 2021, and 2 in 2022). In the included articles, AI was used for 5 purposes: risk prediction, diagnosis, grade prediction, treatment response prediction, and molecular subtype prediction. None of the included articles were used for other purposes such as drug discovery, patient outcomes, and epidemiology. The United States, China, and India (17/30, 57%) were the countries with the highest number of studies related to the use of AI to predict pancreatic cancer. To explore the use of AI technology in the diagnosis of pancreatic cancer, we divided our results into 4 categories, each providing classification of the reviewed publications from a different angle.

The first category focuses on how AI technologies are utilized in the risk prediction and diagnosis of pancreatic cancer and comprises 4 main subcategories: (1) diagnosis of pancreatic cancer using blood samples, gene expression, images, and electronic data; (2) risk prediction of patients using magnetic resonance imaging, disease history, and CT images; (3) differentiation using mRNA and DNA methylation data; and (4) segmentation of CT scan images for diagnosis and prediction. The second category investigates the features of the AI techniques that were present in the studies. Two AI branches were used: general machine learning and deep learning. Deep learning was the most used branch in a total of 11 studies, and the most used model in this branch was the convolutional neural network (18/30, 60%). In contrast, machine learning techniques other than deep learning were used 8 times, and the most used was the regression model (6/30, 20%). The third category focuses on the data used for AI, whereby we classified the data size, data sources, and data types found in the literature for the AI techniques. A total of 28 studies provided the data set size used. The majority of the studies (11/30, 37%) recruited data sets between 100 and 999 samples. K-fold cross-validation was used in 10 (10/30, 33%) studies for AI model testing. Finally, the fourth category classifies the validation method used as well as the statistical data found in the included studies. The included studies used different parameters for statistical validation, including accuracy, sensitivity, specificity, precision, AUC, and *F*_1_-score. The accuracy of the machine learning model ranged from <86% to >98%. Moreover, the sensitivity was only reported in 12 studies, ranging from <90% to >99%, whereas the value of specificity was mentioned in 10 studies. The mean sensitivity was 89.5%, whereas the mean specificity was 89.98%.

### Comparison With Previous Studies

Kenner et al [9] conducted a summative review and provided an overview of AI and early diagnosis of pancreatic cancer. They reported that imaging, blood-based assays, microbiome, and patients’ characteristics can be used for the early diagnosis of pancreatic cancer with the help of machine learning algorithms [9]. Mendoza Ladd and Diehl [46] published a literature review that explored the use of AI techniques in diagnosing pancreatic cancer. They focused on the analysis of endoscopic ultrasound images, CT images, and magnetic resonance images using AI algorithms. Chen et al [47] provided a literature review on the application of AI for pancreatic cancer. They included articles from PubMed and Google Scholar by using the keywords AI and pancreatic cancer and focused on risk assessment, treatment, diagnosis, and prognosis of pancreatic cancer. They did not follow the PRISMA guidelines to ensure the reliability and transparency of the work [47]. Moreover, Dumitrescu et al [10] conducted a systematic review by following PRISMA guidelines and focused on the diagnostic value of AI-based ultrasound for the diagnosis of pancreatic cancer. Bradley et al [11] also followed PRISMA guidelines and critically analyzed the role of machine learning algorithms in clinical decision support systems for pancreatic cancer management. However, this scoping review focuses on the application of AI techniques in the diagnosis, identification, segmentation, and prediction of pancreatic cancer. This is the first review to follow PRISMA guidelines on the AI application for pancreatic cancer diagnosis. Furthermore, this review explores the advantages of different AI techniques for pancreatic cancer. Support vector machine, k-means clustering, and decision tree showed good accuracy for cancer cell differentiation (99%), whereas neural networks that used electronic data for improving the prognosis showed lower accuracy (71.6%).

### Practical and Research Implications

This scoping review explored the AI models and algorithms used in diagnosing and detecting pancreatic cancer. Our findings will assist further research on pancreatic cancer diagnosis. For example, the majority of the studies mentioned reviewed the use of patients’ samples and image data only for pancreatic cancer diagnosis. Hence, future studies should explore the influence of external factors like food, lifestyle, exercise, and environment on cancer patients’ survival and their effects on the performance of AI models.

In this review, most studies used deep learning techniques as compared to other machine learning techniques. It makes sense that most researchers used deep learning methods because they were working with unstructured data. In contrast, structured data, which was less frequently used in the articles under study, work well with some of the other machine learning algorithms. A neural network was the most prominently used algorithm in the included studies, as it can easily work on multidimensional data by using a large set of parameters. Nonetheless, further studies are required on a large set of patients’ data to validate the use of the neural network for pancreatic cancer diagnosis. Moreover, a few studies have discussed the use of machine learning approaches such as logistic regression, random forest, and decision trees, which provide binary classification, suggesting why these methods were adopted.

By correlating the demographic information of the patient with clinical information, pancreatic cancer could be predicted early. The early diagnosis of cancer helps physicians to treat patients effectively and perhaps save their lives. Deep learning models seem to be ideal for data being used (such as CT scan images, electronic data). Nevertheless, future research should focus on genomic and other omics data as well as lifestyle data for diagnosing and predicting pancreatic cancer using AI algorithms.

AI technology requires large data sets to train models; however, here, only 6 studies included used a data set with more than 1000 data points, and other studies mentioned data size as a limitation. Therefore, AI models in future studies should be trained and validated on a larger data set and include healthier and patient samples, as these accounted for less than 30% in the included studies. Also, the hospital systems should use 5G technology and develop a large-scale pancreatic cancer clinical database, which can be used to train AI models for health and medical services [48].

Furthermore, future research should focus on the time period preceding development of pancreatic cancer, during which the patient is at risk of developing the disease, in addition to the percentage of true positive and true negative outcomes. Additionally, more studies are needed to analyze and address hyperparameter optimization because it may affect performance results for AI models in the chosen studies and influence the crucial factors for predicting pancreatic cancer. Further studies should also find new biomarkers for precise prediction of pancreatic cancer at an early stage.

It is also noteworthy that clinical research studies including patients from a single hospital or nation had skewed findings that did not provide true global representation. Thus, future research should consider open data sets that include cases from many hospitals and populations.

**Figure 2:**
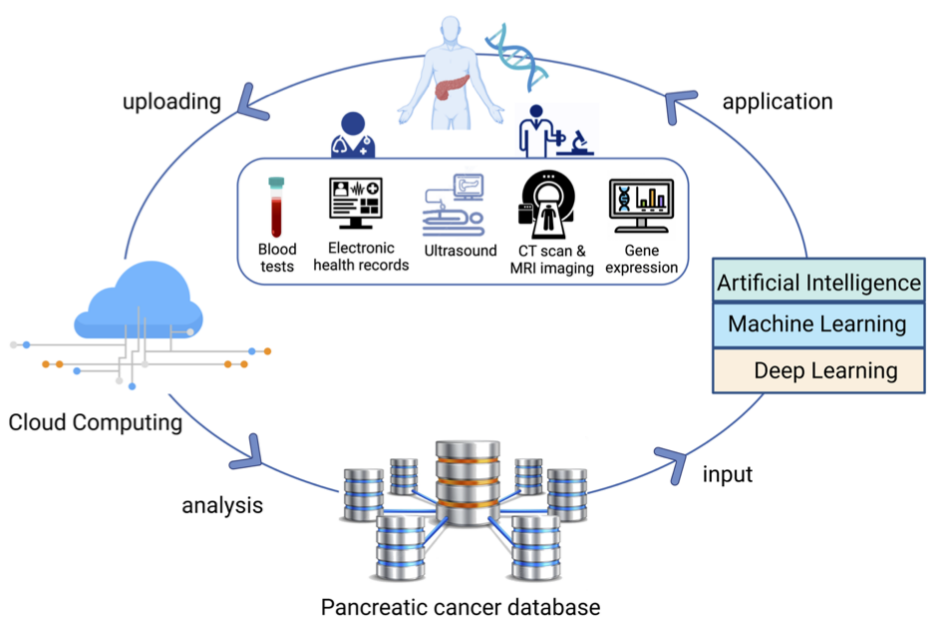
Recommendations for designing an intelligent pancreatic cancer diagnosis and treatment platform. First, upload anonymized patient data from all over the world to a large federated database, and then perform extensive large-scale artificial intelligence (AI) training based on different demographic traits and geographical regions. To improve the current pancreatic cancer treatment methods, the updated and iterative AI system will be used for the precise prediction, screening, diagnosis, and treatment of pancreatic cancer. CT: computed tomography; MRI: magnetic resonance imaging.

Finally, we suggest the development of a network that uses data from different populations of pancreatic cancer patients to develop the database and conduct extensive AI training based on various demographic traits and geographical regions. The exact prevention, screening, diagnosis, and treatment of pancreatic cancer will then be carried out using advanced AI technology, which will help to improve the early diagnosis and treatment of pancreatic cancer. Therefore, in this way, we can develop AI algorithms or models that are appropriate for diagnosing and treating pancreatic cancer and provide solutions for this deadly disease (Figure 2).

### Strengths and Limitations

#### Strengths

Based on our knowledge, this is the first PRISMA-based review to focus on the use of AI techniques for pancreatic cancer. This helps researchers, computer scientists, policy makers, and health care leaders to use AI technology for fighting cancer. The most widely used databases in the information technology and health sector were explored to obtain as many relevant articles as possible. Since there were no restrictions applied to the study design, country of publication, and study settings, this review can be considered inclusive. Additionally, we lessened selection bias by having 2 reviewers independently perform the study selection and data extraction, with perfect agreement in all phases. Moreover, reviewing the data source and platforms used for the AI models and providing the purpose and features of all AI technologies listed in the included studies have strengthened this work.

#### Limitations

This study has the following limitations. Articles related to treatment and drug discovery for pancreatic cancer were not included in this review. Only preprint and journal articles were included, while thesis, review articles, conference abstracts, and review reports were excluded to reduce the complexity of the results. Our included data belonged to 14 countries, as we limited our search to articles from the aforementioned 5 databases. Hence, data from other databases that were published in other countries were not reviewed, potentially reducing the comprehensiveness of our study. Articles published in other languages such as Chinese and French were also not considered. Our search query was not related to specific types of devices, technology, model, or application. Thus, we probably skipped several articles with these terms in their title or abstract instead of using “artificial intelligence.”

### Conclusion

This scoping review was done to support the existing evidence on the role of AI technology in pancreatic cancer diagnosis. We summarized the AI models and algorithms that have been used for prediction and early diagnosis. The use of AI to fight pancreatic cancer is still in its infancy. We believe that this review will help the scientific community to better understand the applications of AI technologies required for risk prediction and diagnosing pancreatic cancer. We also believe that AI techniques offer further unexploited potential in health care for cancer risk prediction and diagnosis, but additional research in this area is needed. For AI to be properly accepted in the real world, standardized protocols to be followed by researchers on AI are required. In addition, more AI ethics research, explainable AI research, and public education initiatives are required.

## Appendix

Multimedia Appendix 1 Search terms used to find studies.

Multimedia Appendix 2 Inter-rater agreement matrices for the study selection.

Multimedia Appendix 3 Description of the data extraction fields.

Multimedia Appendix 4 Summary of the extracted data.

Multimedia Appendix 5 Detailed characteristics of the studies.

Multimedia Appendix 6 Additional detailed characteristics of the studies.

## Abbreviations

AI: artificial intelligence
AUC: area under the curve
CT: computed tomography
PRISMA-ScR: Preferred Reporting Items for Systematic Reviews and Meta-Analyses Extension for Scoping Reviews
