# Supplementary material for: Artificial Intelligence for the Prediction and Early Diagnosis of Pancreatic Cancer: Scoping Review
Source: J Med Internet Res. 2023 Mar 31;25:e44248. doi: 10.2196/44248 (PMC10131763; doi:10.2196/44248)
Supplement: Multimedia Appendix 1 [file jmir_v25i1e44248_app1.docx]

**Appendix 1:** Search Terms used to find studies

| **Database** | **Hits** | **Search Terms** |
| --- | --- | --- |
| PubMed | 60 | ("Artificial intelligence" OR "Machine learning" OR "Deep learning" OR "supervised learning" OR "unsupervised learning" OR "reinforcement learning") AND (“Pancreatic Cancer” OR “Pancreatic adenocarcinoma") AND (diagnos* OR detect* OR predict* OR screen*) |
| BioRXiv | 434 | ("Artificial intelligence" OR "Machine learning" OR "Deep learning" OR "supervised learning" OR "unsupervised learning" OR "reinforcement learning") AND (“Pancreatic Cancer” OR “Pancreatic adenocarcinoma") AND (diagnos* OR detect* OR predict* OR screen*) |
| Google scholar | 17200 | (“Artificial intelligence” OR “Machine learning” OR “Deep learning” OR “supervised learning” OR “unsupervised learning” OR “reinforcement learning”) AND (“Pancreatic Cancer” OR “Pancreatic adenocarcinoma”) |
| MedRxiv | 82 | (“Artificial intelligence” OR “Machine learning” OR “Deep learning” OR “supervised learning” OR “unsupervised learning” OR “reinforcement learning”) AND (“Pancreatic Cancer” OR “Pancreatic adenocarcinoma”) AND (diagnos* OR detect* OR predict* OR screen*) |
| ScienceDirect | 509 | ("Artificial intelligence" OR "Machine learning" OR "Deep learning”) AND (“Pancreatic Cancer” OR “Pancreatic adenocarcinoma") AND (diagnos OR detect OR predict OR screen) |
