# Supplementary material for: Artificial Intelligence for the Prediction and Early Diagnosis of Pancreatic Cancer: Scoping Review
Source: J Med Internet Res. 2023 Mar 31;25:e44248. doi: 10.2196/44248 (PMC10131763; doi:10.2196/44248)
Supplement: Multimedia Appendix 2 [file jmir_v25i1e44248_app2.docx]

**Appendix 2:** Interrater agreement matrices for study selection

| **Interrater Agreement Matrices** | | | | |
| --- | --- | --- | --- | --- |
| **Title and Abstract Screening** | | | | |
|  | | **Reviewer 1** | | |
|  |  | **Include** | **Exclude** | **Total** |
| **Reviewer 2** | **Include** | 390 | 4 | 394 |
|  | **Exclude** | 2 | 778 | 780 |
|  | **Total** | 392 | 782 | **1174** |
| **Full text Screening** | | | | |
|  | | **Reviewer 1** | | |
|  |  | **Include** | **Exclude** | **Total** |
| **Reviewer 2** | **Include** | 30 | 1 | 31 |
|  | **Exclude** | 2 | 357 | 359 |
|  | **Total** | 32 | 358 | **390** |
