# Supplementary material for: Artificial Intelligence for the Prediction and Early Diagnosis of Pancreatic Cancer: Scoping Review
Source: J Med Internet Res. 2023 Mar 31;25:e44248. doi: 10.2196/44248 (PMC10131763; doi:10.2196/44248)
Supplement: Multimedia Appendix 3 [file jmir_v25i1e44248_app3.docx]

**Appendix 3:** Description of the data extraction fields

| **Data Extraction Field** | **Description** |
| --- | --- |
| **Title** | The title of the article |
| **First Author** | The name of the first author in the article |
| **Year of Publication** | The year of the publication of each article was recorded. |
| **Country** | Country in which article published was noted. |
| **Study level** | Studies were conducted at two level.  1: Population level  2: Individual level |
| **Age of the participants** | The age of the participants in the included studies was noted |
| **Participants Health Conditions** | The health conditions of the participants mentioned in the included studies was recorded. |
| **Outcome** | Represent confusion matrix, performance measures (accuracy, sensitivity etc) and statistical validation of the ML models used in the included studies |
| **Data settings** | Represent which type of data utilized by the authors.  Data types was two categories.  1: Clinical  2: Non-clinical |
| **Data Availability** | Represent data are freely available or not.  Two data sources  1: Public- All data used by the author are publicly available  2: Private- Data are not publicly available |
| **AI types** | Show whether included studies used ML or DL or both ML and DL approach |
| **AI model and algorithms** | Represent different model and algorithms used in the included studies |
| **Purpose of AI algorithms** | The purpose of the AI algorithms in the included studies were noted. |
| **Platforms** | Represent which type of platforms were used in the included studies |
| **Programming Languages** | Show which types of programming languages were used in the included articles for data processing and implementation of AI algorithms. |
| **Data type and data size** | Represent data type and data size mentioned in the included studies |
| **Number of features** | Represent how many features are used in the included studies |
| **Publication Type** | Show whether studies are research articles or Conference Proceedings |
| **Study settings** | ML and DL model used in the study was novel or existing. |
