# Supplementary material for: Artificial Intelligence for the Prediction and Early Diagnosis of Pancreatic Cancer: Scoping Review
Source: J Med Internet Res. 2023 Mar 31;25:e44248. doi: 10.2196/44248 (PMC10131763; doi:10.2196/44248)
Supplement: Multimedia Appendix 4 [file jmir_v25i1e44248_app4.docx]

**Appendix 4:** Summary of the extracted data

| **First author** | **Year** | **Country** | **Publication types** | **Publication month** | **Age of the participants** | **Number of participants** | **Gender** | **Participants’ health condition** |
| --- | --- | --- | --- | --- | --- | --- | --- | --- |
| Sadewo et al [17] | 2020 | Indonesia | Journal article | April | NA | 203 | NA | Pancreatic cancer |
| Sinkala et al [16] | 2020 | South Africa | Journal article | January | NA | 185 | NA | Pancreatic cancer |
| Roch et al [18] | 2015 | United States | Journal article | May | NA | 50,669 | NA | Pancreatic cancer |
| Muhammad et al [19] | 2019 | United States | Journal article | January | 40-80 | 800, 114 | Male: 100%  Female: 0% | Pancreatic cancer |
| Alizadeh Savareh et al [20] | 2020 | Iran | Journal article | August | NA | 671 | NA | Pancreatic cancer |
| Mahmoudi et al [21] | 2022 | Iran | Journal article | January | NA | 157 | NA | Pancreatic Cancer |
| Placido et al [22] | 2021 | United States | Journal article | June | 0-120 years old | Danish dataset: 8,110,706 patients.  Boston MGB dataset: 1,015,978 patients; | Males: 45.25%  Female: 54.75% | Pancreatic cancer |
| Turki et al [23] | 2020 | Saudi Arabia | Journal article | January | T2D patients: 37-57 years old.  Healthy donors: 22-48 years old | Dataset of 4 T2D patients; 6 healthy donors | 60% Male  40% Females | Type-2 diabetes (T2D) |
| Liu et al [24] | 2019 | China | Journal article | December | <65 years old: 123 patients.  >65 years old: 215 patients | 338 PC patients | 63% male  37% female | Pancreatic cancer |
| Tonozuka et al [25] | 2021 | Japan | Journal article | September | Mean= 66 years (33-91) | 139 patients: 76 with pancreatic ductal carcinoma, 34 with chronic pancreatitis, and 29 with normal pancreas | 53% male  47% female | Pancreatic cancer |
| Dhruv et al [26] | 2021 | India | Journal article | May | NA | NA | NA | Pancreatic tumor |
| Zhu et al [27] | 2019 | United States | Journal article | October | NA | 439 (303 controls and 136 patients) | NA | Pancreatic cancer |
| Gao et al [28] | 2019 | China | Journal article | September | Mean= 56.52 ± 10.33 | 96 patients | 49% Males  51% Females | Pancreatic tumor |
| Kaissis et al [29] | 2020 | Germany | Journal article | February | 42-90 years | 207 patients | 55% Males  45% Females | pancreatic cancer |
| Dhruv et al [30] | 2022 | India | Journal article | January | NA | NA | NA | Pancreatic tumor |
| Nasief et al [31] | 2019 | United States | Journal article | October | Median= 67 years | 90 PC patients | 54% males and 46% females | Pancreatic cancer |
| Lee et al [32] | 2021 | South Korea | Journal article | September | Median= 65 years | 4846 patients | 58.2% males and females 41.8% | Pancreatic cancer |
| Walczak et al [33] | 2018 | United States | Journal article | December | Mean= 64.47 years | 219 patients | 55.7% males  44.3% females | Pancreatic cancer |
| Luo et al [34] | 2020 | China | Journal article | April | mean= 47.97 (32-66.34) | 112 patients another | 48.4% males and 51.6% females | Pancreatic  cancer |
| Yokoyama et al [35] | 2020 | Japan | Journal article | May | Median=66 years | 191 patients | NA | Pancreatic cancer |
| Sekaran et al [36] | 2019 | India | Journal article | March | 18-76 years old | 80 patients | 66.25% males and 33.75% females | Pancreatic cancer |
| Sala Elarre et al [37] | 2019 | Spain | Journal article | April | Median= 63 years (35-82) | 40 patients | 57.5% males and 17 females 42.5% | Pancreatic cancer |
| Sanoob et al [38] | 2016 | India | Journal article | April | NA | 120 patients | NA | Pancreatic cancer |
| Hsieh et al [39] | 2018 | United States | Journal article | November | T2D patients without PC: mean age of 57.3 years old; T2D patients with PC: mean age of 63.8 years old | 1,358,634 patients | 52.6% males and  47.4% females | Type-2 Diabetes (T2D) |
| Si et al [40] | 2021 | China | Journal article | January | Mean= 63.3 years (37-90 years) | 319 patients | 66.14% males and 33.86 females | Pancreatic cancer |
| Tong et al [41] | 2020 | China | Journal article | March | Mean= 61.11 years (52.5-69.6) | 221 patients | 65.16% males and 34.84% females | Pancreatic cancer |
| Janssens et al [42] | 2021 | United States | Journal article | December | Mean= 52.1 years | 469 patients | 49.7% males and 50.3% females | Pancreatic cancer |
| Chen et al [43] | 2021 | United Kingdom | Journal article | March | Don’t have access | 29,230 patients | Don’t have access | NA |
| Ozkan et al [44] | 2016 | Turkey | Journal article | April | 17-84 years | 172 patients and controls | 49.4% males and 50.6% females | NA |
| Almeida et al [45] | 2020 | Brazil | Journal article | January | NA | 461 tumor and 187 normal samples | NA | Tumor and normal |
