# Supplementary material for: Artificial Intelligence for the Prediction and Early Diagnosis of Pancreatic Cancer: Scoping Review
Source: J Med Internet Res. 2023 Mar 31;25:e44248. doi: 10.2196/44248 (PMC10131763; doi:10.2196/44248)
Supplement: Multimedia Appendix 5 [file jmir_v25i1e44248_app5.docx]

**Appendix 5:** Detailed characteristics

| **Author** | **AI type** | **Algorithm used** | **Statistics** | **Validation method** | **Programming language** | **Data features** | **Data size** | **Purpose** | **Data used** |
| --- | --- | --- | --- | --- | --- | --- | --- | --- | --- |
| Sadewo et al [17] | Machine Learning | twin support vector machine (TWSVM) method | Accuracy= 98%, sensitivity= 97%, specificity=100% | k-Fold Cross Validation | Python3 | blood tests which consisted of cancer antigens, hemoglobin, leukocytes, and platelets | 203 samples | Early diagnosis | Pancreatic cancer data |
| Sinkala et al [16] | Machine Learning | K-means clustering, KNN algorithm, logistic regression, linear discriminate analysis, decision trees, and SVM | Accuracy=99% for mRNA-based KNN classifier and 98% for DNA-methylation based SVM classifier | k-Fold Cross Validation | NA | Protein, mRNA expression data, DNA methylation data | 45 high-purity pancreatic cancer samples | Differentiation | Cancer samples |
| Roch et al [18] | Machine learning | Natural Language Processing | Sensitivity=99.9%, specificity=98.8% | NA | Java | Electronic health record | 50,669 | Early diagnosis | patients |
| Muhammad et al [19] | Machine learning | Neural Network | Sensitivity=87.3 and 80.7%, specificity=80.8 and 80.7%  and AUC=0.86 and 0.85 | 10-fold cross validation | NA | Demographic data | 2 different datasets used:  Set 1: 645217 respondents, 131 of whom had PC  Set 2: 154,897 participants, 767 of whom had PC | Early diagnosis | Normal + patients’ data |
| Alizadeh Savareh et al [20] | Machine learning | Particle Swarm Optimization (PSO), Artificial neural network (ANN) and Neighborhood Component Analysis (NCA) | Accuracy=93%, Sensitivity=93%, Specificity=92% | NA | NA | Gene expression | 671 PDAC patients’ data | Early diagnosis | miRNA |
| Mahmoudi et al [21] | Deep leaning | Deep Convolutional Neural Network | Pre-trained Attention U-Net model precision=76%; Pre-trained TAU-Net model precision=88% and pre-trained hybrid model precision=87% | NA | NA | CT scan images | Set 1: 138 cases  Set 2: 19 cases | Segmentation | Pancreatic cancer |
| Placido et al [22] | Deep learning | Neural network, regression model | Precision=14.0% and specificity=99.7% | NA | NA | Time history of disease | 24, 000 pancreatic cancer | Risk prediction | Patient’s data |
| Turki et al [23] | Deep learning | Deep convolutional neural network (DCNN) | NA | 5-fold-cross validation | Python, R | T2D tissues | 1178 T2D cells and 1313 control cells | Disease differences identification | Patients and healthy controls |
| Liu et al [24] | Artificial intelligence | Region-based convolutional neural network | Precision=76% | NA | NA | CT scan images | 4385 CT images from 238 pancreatic cancer patients | Diagnosis | Patient’s data |
| Tonozuka et al [25] | Deep learning | Convolutional neural network (CNN), Logistic regression analysis | AUC=92%-94& | 10-fold cross validation | NA | Images data | 920 endosonographic images of 139 patients | Diagnosis | PDAC, CP and NP cancer |
| Dhruv et al [26] | Artificial intelligence | Particle swarm intelligence | NA | NA | NA | CT scans images | NA | Diagnosis | images |
| Zhu et al [27] | Deep learning | CNN | Sensitivity=94.1%, Specificity=98.5% | NA | NA | CT scans images | 439 cases | Segmentation | Images |
| Gao et al [28] | Deep learning | Adversarial network (GAN) and a convolutional neural network (CNN) | Accuracy=85.13% and AUC=0.9117 | 5-k cross validation | NA | MR images | 96 patients | Grade Prediction | Private data |
| Kaissis et al [29] | Machine learning | Random Forest | AUC=0.93, Sensitivity=84%, specificity=92% | 5-k cross validation | Python | CT scans images | 207 patients | Molecular subtypes prediction | Private data |
| Dhruv et al [30] | Artificial Intelligence | Partial swarm optimization | NA | NA | NA | CT scans images | 15 images | Diagnosis | CT scans images |
| Nasief et al [31] | Machine learning | Bayesian model | AUC=0.94 | Cross-validation, leave-one-out cross validation, External validation | NA | Images | 90 patients | Treatment response prediction | Images |
| Lee et al [32] | Machine learning | Random forest | AUC=0.68 | Cox model | NA | Real time data of patients | 4846 patients | Prediction | Patient’s data |
| Walczak et al [33] | Deep learning | Neural network | Sensitivity= 91.3% and accuracy=71.6% | Root means square error (RMSE) | NA | Electronic data | NA | Prediction | Electronic data |
| Luo et al [34] | Deep Learning | Convolutional neural network (CNN) | AUC= 0.82 and accuracy=88.1% | cross validation | NA | 112 CT images | 93 patients | Prediction | CT images |
| Yokoyama et al [35] | Machine Learning | Support Vector machine, neural network, multinomial based methods | NA | leave-one-out cross-validation (LOOCV) test | NA | Tissues samples of 191 pancreatic patients | 191 patients | Prediction | Tissues samples |
| Sekaran et al [36] | Deep learning | Deep Convolutional neural network (CNN) | NA | NA | NA | Image data | 19,000 images | Prediction | CT scan images |
| Sala Elarre et al [37] | Machine learning | Logistic regression | Accuracy =95% | k-Cross validation | NA | Real time data of patients | 40 patients | Risk Prediction | Patient data |
| Sanoob et al [38] | Deep learning | Levenberg- Marquardt algorithm, Artificial neural network | NA | Root means square error (RMSE) | NA | Real time data of patients plus control sample | NA | Diagnosis | Patient samples |
| Hsieh et al [39] | Machine Learning | Logistic regression and Artificial neural network | AUC=72.7%, Precision: 99.5%, | k-cross fold validation | Python | 3,092 pancreatic can- cer patients and 1,355,542 nonpancreatic cancer patients | 1,358,634 participants | Prediction | T2DM |
| Si et al [40] | Deep learning | Deep neural network, fully end-to-end deep-learning (FEE-DL) model | Accuracy = 82.7%, specificity = 69.5%,  Sensitivity = 86.8%, AUC= 87.1% | NA | NA | 143,945  CT images | 319 patients | Diagnosis | CT images |
| Tong et al [41] | Machine Learning | Logistic regression and Artificial neural network | AUC = 0.849 | Cross  validation | Python | Clinical and biochemical data | 221 unresectable pancreatic cancer patients | Survival rate prediction | Patient data |
| Janssens et al [42] | Deep learning | Convolutional neural network | NA | NA | NA | CT scans images of patients | 19456 images from 469 non-contrast scans | Diagnosis | CT scans images |
| Chen et al [43] | Machine learning | eXtreme Gradient Boosting (XGBoost) | AUC=0.84, Sensitivity = 60%, specificity =90% | Fold-cross-validation | NA | Electronic health record data | early-stage (n = 3,322) and late-stage (n = 25,908) pancreatic cancer cases | Diagnosis | EHR data |
| Ozkan et al [44] | Machine learning | Neural network | Sensitivity = 93.3%, specificity =94.1%, Accuracy = 92% | NA | NA | Images | 332 endosonography images | Diagnosis | Endosonography images |
| Almeida et al [45] | Machine learning | Neural network | sensitivity = 87.6, specificity = 83.1%,  f1-score= 0.82 | NA | Python | mRNA expression data | 463 tumor samples and 187 normal tissues | Diagnosis | Genes data |
