# Supplementary material for: Artificial Intelligence for the Prediction and Early Diagnosis of Pancreatic Cancer: Scoping Review
Source: J Med Internet Res. 2023 Mar 31;25:e44248. doi: 10.2196/44248 (PMC10131763; doi:10.2196/44248)
Supplement: Multimedia Appendix 6 [file jmir_v25i1e44248_app6.docx]

**Appendix 6:** Detailed characteristics

| **First Author** | **Performance measures** | **Data set source** | **Data input to AI algorithms** | **Number of features** | **Confusion Matrix** |
| --- | --- | --- | --- | --- | --- |
| Sadewo et al [17] | Accuracy, sensitivity, specificity and running time | Closed | Pancreatic cancer | 6 features | NA |
| Sinkala et al [16] | Accuracy, AUC, and Cohen Kappa | Open | Protein, mRNA, methylation, and microRNA | More than 200 | NA |
| Roch et al [18] | True positives, true negatives, false negatives, false positives, sensitivity, and specificity | Closed | EMR | Clinical, radiological, surgical and pathological narrative reports | Yes |
| Muhammad et al [19] | Sensitivity, specificity, and AUC | Closed | Demographic data, Health measures, Physical activity data | 18 features | NA |
| Alizadeh Savareh et al [20] | Accuracy, Sensitivity and Specificity | Open | Microarray datasets | 2 features | Yes |
| Mahmoudi et al [21] | Recall, Precision, DSV | Open | CT scan images | 5 features | NA |
| Placido et al [22] | Precision, Recall, AUC,  F1-scores | Closed | EHR, patient history | 5 features | NA |
| Turki et al [23] | Accuracy (ACC), F1, Matthew’s correlation coefficient (MCC), and area under curve (AUC) | Closed | 224 images of Single-cell pancreatic data of healthy and T2D  pancreas | 7 features | NA |
| Liu et al [24] | True positive rate (TPR), false positive rate (FPR), precision (P) and recall (R) | Closed | CT scan images | 12 features | NA |
| Tonozuka et al [25] | ROC | Closed | Endosonographic images | 2 features | NA |
| Dhruv et al [26] | Entropy | Closed | CT images | 5 features | NA |
| Zhu et al [27] | Sensitivity, Specificity | Closed | CT images | 6 features | NA |
| Gao et al [28] | AUC, Accuracy | Closed | MRI images | 5 features | NA |
| Kaissis et al [29] | Sensitivity, specificity, and ROC-AUC | Closed | CT images | 1474 features | NA |
| Dhruv et al [30] | Entropy | Closed | CT images | 5 features | NA |
| Nasief et al [31] | AUC, ROC | Closed | CT images | 1300 features | NA |
| Lee et al [32] | C-Index | Closed | Patient’s data | 5 features | NA |
| Walczak et al [33] | Sensitivity, Specificity, Accuracy, ROC | Closed | Real time patient data | 14 features | NA |
| Luo et al [34] | AUC, accuracy, sensitivity, specificity, CI, ROC | Closed | CT images | >8,000 radiomics features | NA |
| Yokoyama et al [35] | p-value | Closed | DNA methylation | 9 features | Yes |
| Sekaran et al [36] | NA | Closed | CT images | 4 features | NA |
| Sala Elarre et al [37] | Sensitivity, specificity, and area under the curve (AUC) | Closed | Histological data | 23 features | NA |
| Sanoob et al [38] | Accuracy | Closed | EHR data | NA | NA |
| Hsieh et al [39] | F_1_, precision, recall and ROC | Closed | Demographic data | 4 features | NA |
| Si et al [40] | F_1_, AUC, Accuracy, Sensitivity, Specificity | Closed | CT images | 4 features | Yes |
| Tong et al [41] | AUC, CI, Sensitivity, Specificity, PPV (positive predictive values), PLR (positive likelihood ratio), NPV (negative predictive value) | Closed | Clinical data | 3, 7 and 37 features | NA |
| Janssens et al [42] | p-value | Closed | CT images | NA | NA |
| Chen et al [43] | AUC, PPV, Sensitivity, Specificity, CI | Closed | EHR data | 18,220 features | NA |
| Ozkan et al [44] | Accuracy, Sensitivity, Specificity | Closed | Endosonography images | 20 features | NA |
| Almeida et al [45] | F1-score, Sensitivity, sensibility, and accuracy | Open | mRNA expression | NA | Yes |
